# Latin American Network for Scientific Culture (RedLCC): A Regional Science Communication Initiative

**DOI:** 10.3389/frma.2021.654022

**Published:** 2021-03-23

**Authors:** Felix Moronta-Barrios, Santiago Vargas-Domínguez, Melanie Nuesch-Germano, Vicente Torres, Katherina Selvaggi, Cecilia Di Prinzio, Emma O'Brien, Victor Hernandez, Martin Monteiro

**Affiliations:** ^1^International Centre for Genetic Engineering and Biotechnology, Trieste, Italy; ^2^Universidad Nacional de Colombia, Observatorio Astronómico Nacional, Bogota, Colombia; ^3^Life and Medical Sciences Institute, University of Bonn, Bonn, Germany; ^4^Faculty of Medicine, National Autonomous University of Mexico, Mexico City, Mexico; ^5^University School of Medical Technology, University of the Republic, Montevideo, Uruguay; ^6^AcercaCiencia, Rosario, Argentina; ^7^Faculty of Sciences, National Autonomous University of Mexico, Mexico City, Mexico; ^8^School of Engineering, Universidad ORT Uruguay, Montevideo, Uruguay

**Keywords:** Science communication, Scientific culture, Science diplomacy, Science communication in the developing world, Public understanding of science and technology, Public engagement with science and technology, Popularization of science and technology

## Introduction

Scientific communication has been growing strong worldwide in the past decades. The use of modern data analysis tools to fine-tune its content, strategy, and effectiveness, together with the significative rise of social media, have contributed to such significative growth (Kappel and Holmen, 2019). Social media (such as blogs and microblogging) are powerful engines greatly incorporated into our daily lives for capturing information and as a social tool. As such, they are already being exploited for learning, discovering, searching, storing, and sharing knowledge (López-Goñi and Sánchez-Angulo, 2018). Research has shown that online media use increases scientific knowledge (Cacciatore et al., 2014; Su et al., 2015) and positive attitudes toward science (Dudo et al., 2011) therefore enhancing the learning and science process skills. Such scientific knowledge is a critical resource that enables political actors to inform and legitimate political decisions, and it is also important for non-scientific audiences in terms of forming public opinion about important political issues (Huber et al., 2019). Moreover, previous work has demonstrated that democratic societies that are scientifically literate make equitable choices regarding science-related policy issues (European Commission, 1995; Rudolph and Horibe, 2016). Thus, according to Márquez and Porras (2020), effective science communication and science literacy are socioeconomically imperative for all societies. At the same time, Science Communication can serve various diplomatic purposes. Particularly, science popularization initiatives, even when not targeted to policymakers or diplomats, can both raise awareness about international scientific cooperation and about locally produced science and technology which could be highly overlooked (Leach, 2015).

English is currently the lingua franca of science. Currently, 98% of publications in science are written in English (Ramírez-Castañeda, 2020). This has facilitated the dissemination of knowledge across boundaries, but at the same time, the hegemony of English in science promotes and enforces the imposition of just one cultural point of view over others (Márquez and Porras, 2020). Because of that, generating science communication multilinguistic alternatives promotes diversity and creates culturally relevant content.

Science communication in Spanish is especially imperative in Latin America. The intrinsic functional illiteracy, framed in the lack of economic and educational resources, inequality, poverty, political and social instability, are historical challenges that keep this region from unleashing its full potential (UNESCO, 2020). Moreover, and due to these issues, Latin America faces a human capital flight crisis, in which a high percentage of the individuals pursuing higher academic education end up emigrating and learning a second language. This is reflected in a marked lack of availability of educational resources in Spanish addressed to Latin American communities.

Nevertheless, there have been efforts to build remote networks of Latin American scientists and science communicators that come together to counteract this effect. Three renown projects, different in nature, can be used as examples. First, the RedPop (Latin American and Caribbean Network for the Popularization of Science and Technology https://www.redpop.org) is a network of centers and programs created in 1990 at the request of UNESCO's program for Science, Technology and Society (Massarani et al., 2015). It encompasses around 80 science communication projects in different media platforms, but also science museums, interactive science centers, natural history museums, environmental parks, zoos, botanical gardens, and aquariums. Second, the bilingual science communication portal Latin American Science (www.latinamericanscience.org) publishes pieces written by scientist and science writers for the public both in Spanish and English-speaking countries. It focuses on regionally produced research, science policy and science-related stories from the region. And third, the Journal of Science Communication JCOM América Latina (https://jcomal.sissa.it/jcomal/index.jsp), an open access journal focused on science communication in Latin América and publishing contributions in Spanish and Portuguese (Weitkamp and Massarani, 2018). Still, more opportunities need to exist in term of communicating science with regional relevance.

Blogs and social media platforms, which are especially open and easily accessible resources, have fantastic potential to address this gap since it is allowing information and education to reach every home to an unprecedented extent. One of these regional initiatives, the Latin American Network for Scientific Culture (RedLCC), brings together regional scientists that communicate science for Latin American communities and, in consequence, also nurtures the “Science for Diplomacy” dimension of Science Diplomacy.

## The Latin American Network for Scientific Culture

The RedLCC (by its acronymous in Spanish Red Latinoamericana de Cultura Científica) gathers efforts for the dissemination of science and technology carried out by a group of Latin American scientists. They aim to serve as a link between scientific activity and society. As a committed, professional, multidisciplinary, and multinational initiative, the Network offers quality and culturally relevant scientific information for the non-scientific audiences in Latin America. From their different disciplines and concerns, the scientific communicators intend to restore the value of science as a fundamental part of the Latin American human cultural heritage.

The RedLCC's original founders virtually met for the first time when contributing to a Twitter hashtag in July 2012. Their common interest and passion for science communication using blogs, motivated them to join efforts and create the foundational Latin American Network of Scientific Blogs. Based on agreed statutes, which ruled the accession, permanence, and the task distribution among the members, the Network grew and agglutinated up to 22 Latin American scientific blogs within the following few years.

By November 2016, the tremendous growth of members, joining requests, and especially the raising of alternative science communication channels and web resources beyond blogging, drove a re-foundation of the Network. To adapt to the new digital environment in science communication the Network's mandate was changed, and the Latin American Network for Scientific Culture was established. Once the statutes were accordingly updated, not only blogs but other science communication channels can be part of the Network. As such, the Network expanded and diversified its information format and structure.

**Figure 1:**
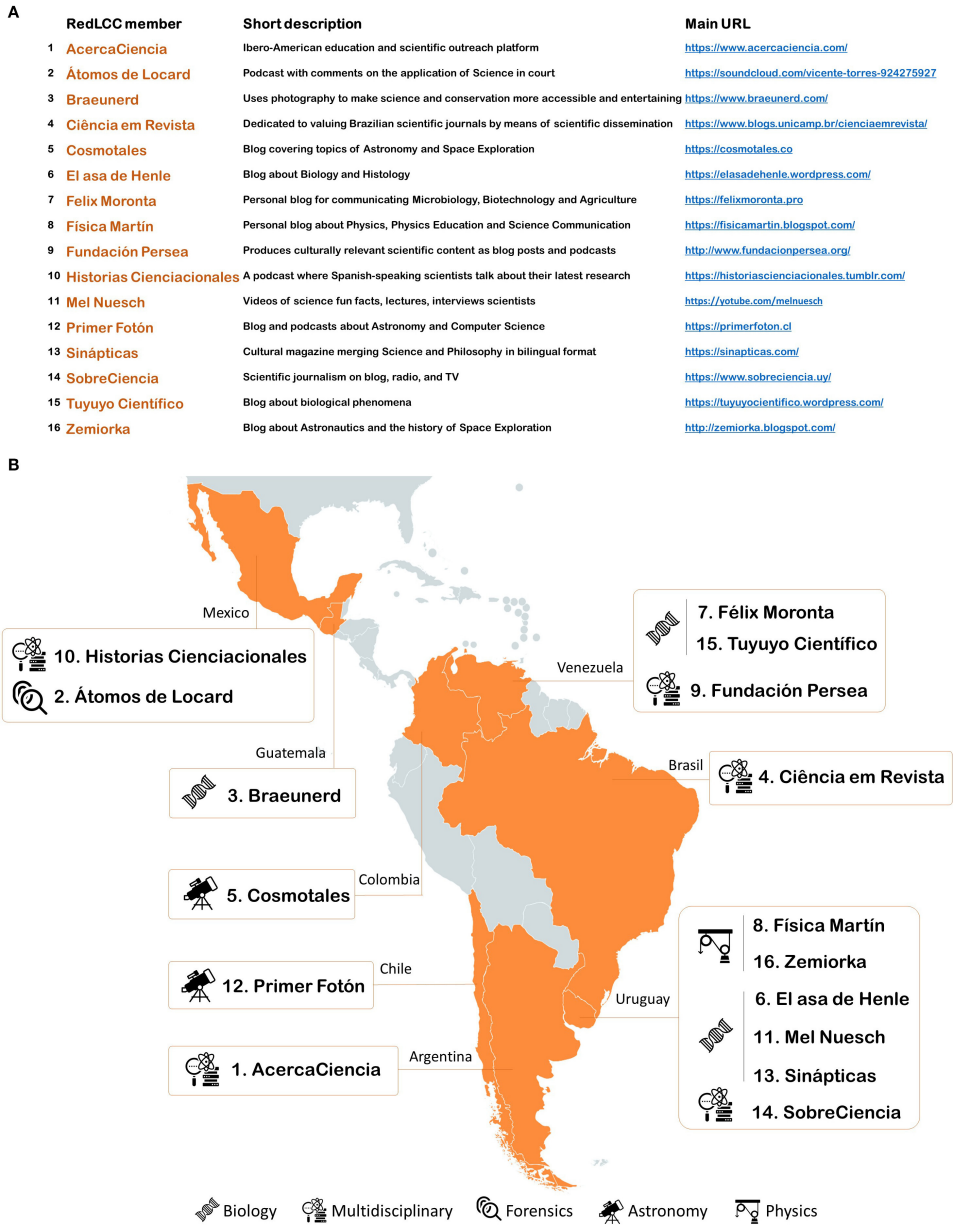
The Latin American Network for Scientific Culture (RedLCC). **(A)** List of the 16 members that compose the Network, in alphabetical order, by December 2020. A brief description of each one and the URL for accessing their content are shown. **(B)** The geographical origin of each of the initiatives with their scientific disciplines.

The RedLCC is a regional, multidisciplinary, and grassroots initiative that brings science to the non-scientific public. To date, the Network is voluntarily composed of 16 initiatives led by scientists from eight countries (Figure 1), who recognize the cultural background of their target community. Therefore, the RedLCC members employ culturally relevant expressions, metaphors, and storytelling approaches for creating emotional connections and engaging with various audiences. With this strategy to support science dissemination efforts in languages other than English, this Latin American initiative could effectively lower the barriers of access to knowledge and promote the interest in science.

There is a growing community around the RedLCC. The Network's blog (http://www.redlcc.org/) accumulates 116,670 visits from 109 countries to date, being 2020 the year with more visitors (an average of 80 daily visits). These numbers are greatly surpassed if the individual statistics of members are considered: a total of 1,435,404 accumulated visits are reported by all members. On the other hand, on Twitter (where all members are present and firmly active) @RedLCC is followed by 4,013 users, of which the 97.1% are real followers. It is estimated that the 58.3% are based in the Americas and Spain, the 64.4% uses Spanish as their primary language, and that for each woman follower there are 1.7 men followers. These metrics inform about the trust in the Network and serve for future improvements in the communication strategies.

The RedLCC provides direct access to scientific information directly posted by trusted scientists, strengthening public engagement with news posted by people they trust. Audiences are more likely to trust and share science news and content on social media because they are posted by a reliable source, helping also to prevent fake news that misinforms or deceive readers. By bringing together trusted communicator scientists in one place, the RedLCC's members are constructing a reference platform in the region for strengthening a regional scientific literacy, also nurturing the traditional communication channels in our region and the dynamics that are commonly used in journalism. Especially during the current COVID-19 crisis, the need for having robust, fast-responding networks to provide high-quality scientific information, news and educational content via online services has been proven to be of the utmost importance, not only to synchronize people's actions and efforts to work together toward maintaining safe public health, but also to highlight the crucial role of scientific research and its global impact on society.

Taking advantage of the increased connectivity of millions of citizens during 2020, new initiatives have emerged that articulate a common interest of our members to strengthen their relationship with different non-scientific actors in society. By using mass dissemination channels as well as webinar platforms, members reported the organization of live streaming events and conferences (e.g., Ciencia Viral, Maratón Cósmico, or COVID-related webinars) to reach those citizens eager for accurate and reliable information.

It is important to underline that the scientific communication activities carried out by the Network's members are not based on digital media only. Many of them report several local activities in their communities. Scientific demonstrations in schools or public places, interviews in local media (TV, radio, and newspapers), astronomical events, master classes, or participation in the editing of school and university texts are part of these activities. These offline actions often have a profound impact on the non-scientific public that has not been engaged with the Network digital media.

Together with the other regional initiatives (such as RedPOP, LatinAmericanScience, or JCOM América Latina), the RedLCC has emerged and established as a reliable source of scientific information and scientific engagement with culturally relevant content. As such, it could also inform policy-related areas about relevant scientific outputs from the region. In the light of the Sustainable Development Goals (SDGs), the Network is taking actions to address directly specific targets within the SDG #4 Quality Education, and indirectly transversal targets.

The role of the RedLCC in Science Diplomacy can be characterized by a framework that describes the contribution of science popularization initiatives to diplomacy (Leach, 2015). Not all science communication projects need to engage with policymakers or with diplomats for them to contribute to soft powers in the region. Specifically, we identify that our work within the RedLCC can serve two diplomatic purposes. First, it raises awareness of outcomes of large-scale international projects, and, mainly, of the participation of Latin American researchers in these projects. Second, it encourages high levels of scientific literacy, awareness, and dialogue among Latin American non-scientific audiences, specially about the scientific work made by regional scientists. These activities leverage public support for regionally produced science and technology, which in turn can be used by regional institutions as a “Science for Diplomacy” tool to advocate for Science Diplomacy.

## Concluding Remarks

The RedLCC is a coordinated and organized regional effort lead by Latin American scientists for strengthening the scientific culture in the region. It is a social-oriented initiative that makes science accessible for many citizens linked to online social media. By communicating the findings, methods, or nature of research to audiences other than the scientific community, the Network encourages high levels of general scientific literacy, awareness, and dialogue about science and technology in Latin America. Therefore, citizens and political actors are empowered to make inform decisions. Consequently, the RedLCC encourages and reinforces agendas of science cooperation backed by both the non-specialized public and the scientific community engaged in science communication. Considering the regional and global challenges to be addressed, especially under the beginning of the Decade of Action to deliver the SDGs, the RedLCC acquires a key role that could contribute not only to enhance the scientific culture in the region but also nourishing soft power resources that have the potential to produce diplomatic outcomes.

## Author Contributions

FM-B conceived the presented Opinion article. All authors discussed the content and contributed to the final manuscript.

## Conflict of Interest

The authors declare that the research was conducted in the absence of any commercial or financial relationships that could be construed as a potential conflict of interest.
